# Association between left ventricular diastolic function and right ventricular function and morphology in asymptomatic aortic stenosis

**DOI:** 10.1371/journal.pone.0215364

**Published:** 2019-07-30

**Authors:** Nicolaj Lyhne Christensen, Jordi Sanchez Dahl, Rasmus Carter-Storch, Kurt Jensen, Redi Pecini, Flemming Hald Steffensen, Eva Vad Søndergaard, Lars Melgaard Videbæk, Jacob Eifer Møller

**Affiliations:** 1 Department of Cardiology Odense University Hospital, Odense, Denmark; 2 Department of Sports Science and Clinical Biomechanics University of Southern Denmark, Odense, Denmark; 3 Department of Cardiology, Lillebaelt Hospital, Vejle, Denmark; Indiana University, UNITED STATES

## Abstract

**Background:**

Aortic stenosis (AS) is a progressive disease in which left ventricular (LV) diastolic dysfunction is common. However, the association between diastolic dysfunction and right ventricular (RV) loading conditions and function has not been investigated in asymptomatic AS patients.

**Methods and findings:**

A total of 41 patients underwent right heart catheterization and simultaneous echocardiography at rest and during maximal supine exercise, stratified according to resting diastolic function. Cardiac chamber size and morphology was assessed using cardiac magnetic resonance imaging (cMRI). RV stroke work index, pulmonary artery (PA) compliance, PA elastance, PA pulsatility index, and right atrial pressure (RAP) were calculated at rest and maximal exercise. Ten patients (24%) had normal LV filling pattern, 20 patients (49%) had grade 1, and 11 patients (27%) had grade 2 diastolic dysfunction. Compared to patients with normal diastolic filling pattern, patients with diastolic dysfunction had lower RV end-diastolic volume (66 ± 11 ml/m^2^ vs. 79 ± 15 ml/m^2^, p = 0.02) and end-systolic volume (25 ± 7 ml/m^2^ vs. 32 ± 9 ml/m^2^, p = 0.04). An increase in mean RAP to ≥15 mmHg following exercise was not seen in patients with normal LV filling, compared to 4 patients (20%) with mild and 7 patients (63%) with moderate diastolic dysfunction (p = 0.003). PA pressure and PA elastance was increased in grade 2 diastolic dysfunction and correlated with RV volume and maximal oxygen consumption (r = -0.71, p < 0.001).

**Conclusions:**

Moderate diastolic dysfunction is associated with increased RV afterload (elastance), which is compensated at rest, but is associated with increased RAP and inversely related to maximal oxygen consumption during maximal exercise.

## Introduction

Calcified aortic stenosis (AS) is a chronic, slowly progressing disorder where pressure overload can lead to heart failure symptoms warranting aortic valve replacement [1]. However, some patients remain asymptomatic for years with gradual adaptation to pressure overload. Typically, the adaptive response will counterbalance pressure overload, in which left ventricular (LV) hypertrophy will help maintain end-systolic wall stress and preserve adequate cardiac output. However, adaptive concentric remodeling over time is associated with diffuse fibrosis and impaired coronary flow reserve, leading to increased myocardial stiffness and diastolic dysfunction [2–4]. The filling pressure increases during diastolic dysfunction especially during exertion, thereby exposing the right ventricle (RV) to increased afterload through the pulmonary vascular bed [5]. Prior hemodynamic studies have shown that loading conditions on the left side of the heart inevitably affects RV diastolic properties and pressure, possibly through ventricular interdependence [6]. Further, previous studies have demonstrated that exercise in symptomatic as well as asymptomatic patients with AS inflicts severe post-capillary pulmonary artery hypertension, thus exposing the RV to increased afterload [7, 8].

There are no previous studies that have assessed the association between LV diastolic dysfunction and RV physiology and morphology in a population with asymptomatic AS. Thus, it is not known whether LV diastolic dysfunction causes alterations in RV function, and whether this is due to ventricular interdependence or chronically elevated LV filling pressures causing afterload mismatch. The objective of this study was to investigate the association between LV diastolic function and RV morphology and function at rest and during exercise in asymptomatic patients with significant AS.

## Methods

A total of 41 patients with significant AS defined as aortic valve area (AVA) < 1cm^2^, aortic peak velocity > 3.5 m/s and LV ejection fraction > 50% were prospectively enrolled from January 2014 to January 2016.

Initially, a total of 215 patients were screened. However, 174 patients were excluded due to s-creatinine > 200 μmol/l (n = 9), pacemaker dependency (n = 11), more than mild co-existing valve disease (n = 11), chronic atrial fibrillation (n = 16), inability to perform exercise testing (n = 25), LVEF < 50% (n = 8), and no consent (n = 94). Informed written consent was obtained at inclusion, and patients were carefully questioned to rule out any symptoms. Of the remaining 41 patients, 39 patients have been reported in a previous study [7]. After completion of that study, 2 additional patients were enrolled in the present study.

All patients underwent right heart catheterization at rest and during multistage symptom-limited exercise testing with simultaneous echocardiography. Cardiac magnetic resonance imaging (cMRI) and cardiopulmonary exercise testing were performed at different timepoints.

The study was registered at the Danish Data Protection Agency and ClinicalTrials.gov (NCT02395107) and approved by the local ethics committee (S-20130067). All patients provided informed consent before enrollment.

### Echocardiography

Transthoracic echocardiography was performed and stored for later blinded analysis on a Vivid E9 (General Electric, Horten, Norway) ultrasound system. An average of 3 to 5 heartbeats was measured for Doppler recordings. Frame rate was kept at a minimum of 60 sec^-1^. The measurements were standardized to body surface area, as appropriate.

Maximal aortic valve jet velocity was assessed in multiple apical views using continuous wave (CW) Doppler. Left ventricular outflow tract (LVOT) diameter was measured 5 mm below the aortic valve. AVA was calculated using the continuity equation.

Left atrium (LA) endocardial border was traced in apical 4 and 2 chamber views, and LA volume was calculated using the biplane method of disks (modified Simpson's rule) and standardized to BSA [9].

Mitral inflow pattern was assessed with the pulsed-wave (PW) Doppler sample volume placed at the tips of the mitral valve leaflets during diastole. From mitral inflow, the peak early (E) and late (A) wave velocity, as well as mitral E-wave deceleration time were measured. Myocardial velocity was recorded using tissue Doppler imaging with a pulsed wave sample volume placed in the medial mitral annulus. From these recordings, tissue Doppler early diastolic (e’) velocity was measured at the septal mitral annulus. Diastolic function was graded based on: E/A ratio, e’, E/e’ ratio, and LA volume index [10]. Patients with EA-ratio 0.8 to 2, e’ > 7 cm/s, and LA volume index < 34 ml/m^2^ were considered to have normal diastolic function. Patients with E/A ratio <0.8 and e’ ≤ 7 cm/s were considered to have grade 1 diastolic dysfunction, and patients with EA-ratio 0.8 to 2, LA volume index ≥ 34 ml/m^2^, and e’ ≤ 7 cm/s were considered to have grade 2 diastolic dysfunction.

### Cardiac magnetic resonance imaging

cMRI was performed on a Philips Ingenia 1.5T scanner with Omega HP gradient system (Philips Electronics, Koninklijke, Netherlands). Images were analyzed by an experienced examiner blinded to both the invasive and echocardiographic data on a dedicated work station with a Philips Intellispace software package (2.6.3.5 2013).

Atrial and ventricular chamber volumes and LV mass were measured from a cine sequence with continuous short axis slices covering the atria and ventricles. LV mass was calculated at end-diastole as the inter-slice gap multiplied by the difference between epicardial and endocardial traced areas with a myocardial density of 1.05 g/ml [11]. Papillary muscles were considered to be part of the LV cavity and excluded from LV mass, and the left atrial (LA) appendage volume was included in the LA volume [12]. The RV was manually traced up to the pulmonary valve and trabeculations, and papillary muscles were included in RV volumes. RV volume in end-diastole and end-systole, RV ejection fraction, and RV stroke volume were measured. Late gadolinium enhancement imaging was performed 10–15 minutes after administration of 0.1 mmol/kg gadoterate meglumine (Dotarem, Guerbet, Aulnay-Bois, France). Late enhancement pattern was reported as midwall, ischemic, and “non-specific”. Radial and longitudinal distribution as well as segment thickness was recorded and counted using a 17 segment cardiac model [13].

### Right heart catheterization

Right heart catheterization was done with a standard 7.5-F triple lumen Swan-Ganz catheter (Edward Lifesciences, Irvine, CA) advanced to the pulmonary artery. Patients underwent a multistage symptom-limited exercise test from rest until exhaustion with increments of 25 Watts every 3 minutes with the catheter in place. Exercise tests were done using an exercise bike (Echo Cardiac Stress Table, Lode B.V., The Netherlands) suitable for exercise echocardiographic studies with an electrical adjustable slope, and the patients were tested in a semi-supine position.

Pulmonary artery wedge pressure (PAWP) was measured at end-expiration during rest and averaged for > 10 secs, whereas mean PAWP was measured during exercise. Blood pressure, heart rate, and arterial oxygen saturation were monitored continuously. Further, right atrial pressure (RAP), pulmonary artery pressure (PAP), and 3 consecutive measurements of cardiac output with <10% variability using thermodilution were recorded at each exercise level. Oxygen saturation and lactate concentration were measured in a mixed central venous blood sample before and immediately after termination of exercise.

### Hemodynamic calculations

RV stroke work index (RVSWi) was calculated using the formula: RVSWi = 0.0136*stroke volume index (SVi)*(mean PAP-RAP) [14]. Pulmonary artery (PA) compliance was calculated using the formula: SV*(systolic PAP-diastolic PAP)/RAP [15] and PA elastance was calculated as systolic PAP/SV [16]. Further, RAP/PAWP ratio and PA pulsatility index ((systolic PAP-diastolic PAP)/RAP) was calculated. In addition, valvuloarterial impedance (Z_va_ = (systolic arterial pressure + mean transvalvular pressure gradient)/stroke volume index) [17]. Calculations were performed at rest and at maximal exercise capacity.

### Maximal oxygen consumption

Thirty-one patients underwent a standard cardiopulmonary Bruce treadmill test to determine maximal oxygen consumption [18]. Respiratory variables (volume of oxygen consumption, volume of carbon dioxide, and heart rate) were measured continuously during the test using an online metabolic unit (Amis 2001; Innovision, Odense, Denmark). Maximal oxygen uptake was measured at the highest value > 30 seconds periods during the last part of the test.

### Statistical analysis

Data are presented as mean ± standard deviation or median (interquartile range) unless otherwise indicated. Between group differences for continuous variables with a Gaussian distribution were tested using ANOVA. For non-Gaussian distributed variables, a nonparametric rank-sum test was used. Fisher’s exact test was used to compare proportions of categorical variables. Linear bivariate analysis was assessed using Pearson’s correlation coefficient. Multiple regression analysis was used to evaluate associations between invasive hemodynamic calculations and oxygen consumption. A p-value of < 0.05 was used as the cutoff for statistical significance on a two-tailed test. STATA/SE 14.0 (StataCorp LP, Texas) software was used for statistical analysis.

## Results

All 41 patients completed the exercise protocol and 1 patient did not complete cMRI due to claustrophobia. Patients were classified into 3 groups according to LV diastolic filling pattern: 10 patients (24%) had normal diastolic filling pattern, 20 patients (49%) had grade 1 diastolic dysfunction, and 11 patients (27%) had grade 2 diastolic dysfunction. Grade 3 diastolic dysfunction was not observed in this cohort. Patients with diastolic dysfunction were older compared to those with a normal filling pattern (p<0.001) (Table 1). Further, during Valsalva maneuver, E/A ratio decreased significantly in patients with moderate diastolic dysfunction (0.71 ± 0.21 vs. 0.59 ± 0.17, p<0.001). The severity of AS in terms of AVA (p = 0.76), mean aortic gradient (p = 0.73), or aortic peak gradient (p = 0.77) was unrelated to LV diastolic function. Resting Z_va_ was significantly higher in patients with diastolic dysfunction compared to those with a normal diastolic filling pattern (3.98 ± 0.61 vs. 4.62 ± 0.88 mmHg*ml^-1^*m^-2^, p = 0.04), which was driven by patients with grade 1 diastolic dysfunction (Table 1). Although TAPSE (tricuspid annular plane systolic excursion) was numerically higher in patients with grade 2 diastolic dysfunction, no significant difference was observed between groups (p = 0.19). However, S’ measured in the basal RV free wall was higher in patients with moderate diastolic dysfunction compared to those with normal diastolic function (Table 1).

**Table 1:** Demographic data and selected echocardiographic parameters according to LV diastolic function.

	All patients n = 41	Normal n = 10	DD-1 n = 20	DD-2 n = 11	P-value* ANOVA
**Age (years)**	73 ± 8	64 ± 9	76 ± 6	73 ± 7	<0.001
**Sex, male (%)**	30 (73)	6 (60)	15 (75)	9 (82)	0.54
**BSA (m^2^)**	1.90 ± 0.19	1.89 ± 0.21	1.87 ± 0.15	1.98 ± 0.22	0.28
**Hypertension (%)**	29 (71)	5 (50)	14 (70)	10 (91)	0.14
**Diabetes (%)**	5 (12)	0 (0)	3 (15)	2 (18)	0.57
**E-GFR (ml/min)**	75 ± 16	77 ± 16	71 ± 17	78 ± 15	0.48
**AVA (cm^2^)**	0.82 ± 0.17	0.80 ± 0.14	0.82 ± 0.18	0.85 ± 0.18	0.76
**AV Vmax (m/s)**	4.2 ± 0.5	4.1 ± 0.5	4.3 ± 0.6	4.2 ± 0.5	0.77
**AV MG (mmHg)**	45 ± 13	42 ± 12	46 ± 14	46 ± 11	0.73
**E’ septal (cm/s)**	0.06 ± 0.02	0.08 ± 0.02	0.06 ± 0.01	0.06 ± 0.01	0.006
**E/e’ (cm/s)**	12 ± 4	11 ± 3	12 ± 4	15 ± 5	0.02
**DT (msec)**	302 ± 96	211 ± 27	376 ± 85	252 ± 33	<0.001
**E velocity (m/sec)**	0.72 ± 0.20	0.80 ± 0.22	0.64 ± 0.19	0.81 ± 0.17	0.03
**A velocity (m/sec)**	0.96 ± 0.30	0.73 ± 0.14	1.05 ± 0.34	1.02 ± 0.22	0.02
**EA ratio**	0.79 ± 0.25	1.09 ± 0.24	0.62 ± 0.10	0.82 ± 0.20	<0.001
**TAPSE (mm)**	23 ± 3	22 ± 2	23 ± 2	24 ± 4	0.19
**S’ RV (cm/s)**	0.13 ± 0.03	0.12 ± 0.01	0.13 ± 0.02	0.15 ± 0.03	0.04
**GLS (%)**	-17.6 ± 2.3	-19 ± 2.6	-17 ± 2.0	-18 ± 2.2	0.15
Zva (mmHg*ml^-1^*m^**-2**^)	4.5 ± 0.9	4.0 ± 0.6	4.9 ± 0.8	4.2 ± 0.8	0.007

Data are shown as number (%) or mean±SD.

BSA = body surface area; E-GFR = glomerular filtration rate; LAVi = LA volume index; AVA = aortic valve area; AV V_max_ = aortic valve Vmax; AVMG = aortic valve mean gradient; DT = deceleration time; TAPSE = tricuspid annular plane systolic excursion; GLS = global longitudinal strain; Valsalva = Valsalva maneuver; Z_va_ = valvulo-arterial impedance.

*ANOVA or Fisher’s exact test for parameters according to diastolic dysfunction (0/1/2).

### Cardiac magnetic resonance

**Fig 1:**
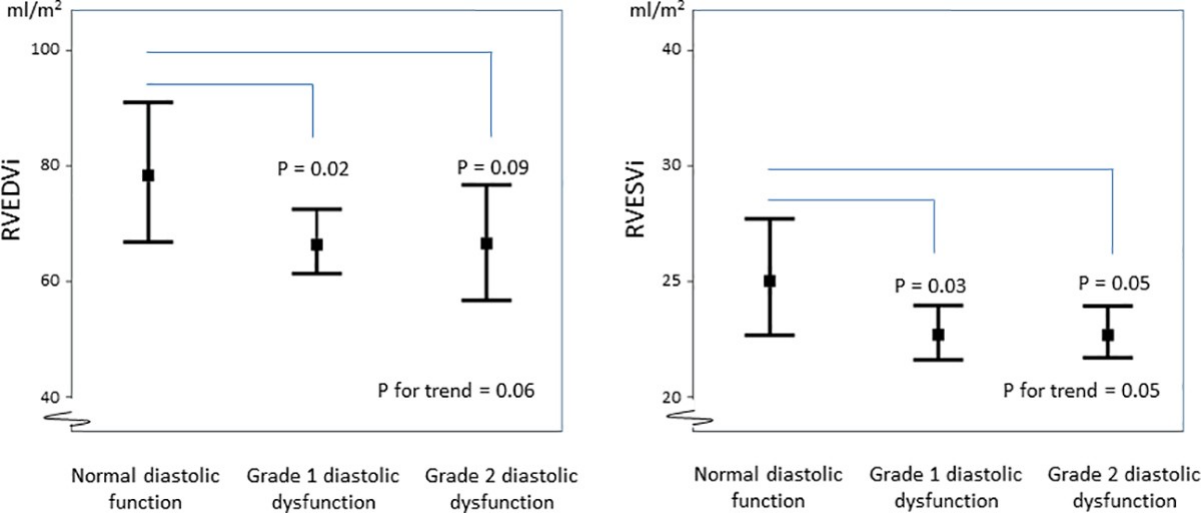
RV end-diastolic and end-systolic volume indices according to LV diastolic function. RVEDVi = right ventricular end-diastolic volume index, RVESVi = right ventricular end-systolic volume index.

Compared to patients with normal diastolic filling pattern, patients with diastolic dysfunction had lower RV end-diastolic volume (66 ± 11 ml/m^2^ vs. 79 ± 15 ml/m^2^, p = 0.02) and end-systolic volume (25 ± 7 ml/m^2^ vs. 32 ± 9, p = 0.04) (Table 2 and Fig 1)). In addition, patients with abnormal LV filling had lower RV pulmonary stroke volume index (40 ± 8 ml/m^2^ vs. 47 ± 9 ml/m^2^, p = 0.02). No association between RV ejection fraction (RVEF) and diastolic function was observed (p = 0.55). In patients with diastolic dysfunction, no differences in LV size, mass, or LVEF were found (Table 2). On LGE, 10 patients (25%) had evidence of LV myocardial replacement fibrosis but was not associated with the diastolic function (p = 0.40). Seven patients presented with midwall fibrosis, 2 had a non-specific fibrotic pattern, and 1 patient had an ischemic pattern.

**Table 2:** Cardiac magnetic resonance imaging and hemodynamic parameters according to LV diastolic function. Demographic and echocardiographic parameters.

	All patients n = 40	Normal n = 10	DD-1 n = 19	DD-2 n = 11	P-value* ANOVA
**RAVi (ml/m^2^)**	51 ± 11	54 ± 7	49 ± 11	52 ± 15	0.45
**RAVi min (ml/m^2^)**	31 ± 9	32 ± 6	29 ± 8	33 ± 11	0.38
**RA emptying fraction, (%)**	40 ± 10	42 ± 8	42 ± 10	36 ± 10	0.27
**RVEDVi (ml/m^2^)**	69 ± 14	79 ± 15	66 ± 11	67 ± 14	0.06
**RVESVi (ml/m^2^)**	27 ± 8	32 ± 9	25 ± 7	25 ± 6	0.05
**RV stroke volume index (ml/m^2^)**	42 ± 8	46 ± 8	41 ± 7	42 ± 11	0.27
**RV ejection fraction (%)**	61 ± 6	59 ± 7	62 ± 6	62 ± 6	0.55
**LV EDVi (ml/m^2^)**	86 ± 18	90 ± 14	83 ± 19	87 ± 18	0.60
**LV ESVi (ml/m^2^)**	33 ± 11	31 ± 7	33 ± 13	33 ± 11	0.05
**LVMi (g/m^2^)**	73 ± 17	75 ± 19	71 ± 19	75 ± 13	0.76
**LV ejection fraction (%)**	62 ± 7	65 ± 4	61 ± 8	62 ± 7	0.32

Data are shown as mean±SD.

DD = diastolic dysfunction; EDVi = end-diastolic volume index; ESVi = end-systolic volume index; LA = left atrial; LV = left ventricular; RA = right atrial; RAVi = right atrial volume index; RV right ventricular

### Invasive hemodynamics

Diastolic dysfunction was associated with increased systolic and diastolic PAP, whereas no difference in CI was found at rest, and there was no difference in SVR at rest (Table 3). Driven by the increased PAP, PA compliance was reduced in patients with diastolic dysfunction (5.6 ± 1.1 ml/mmHg vs. 7.1 ± 2.7 ml/mmHg, p = 0.05) while the PA elastance increased (0.38 ± 0.09 mmHg/ml vs. 0.28 ± 0.08 mmHg/ml, p = 0.005). RV stroke work was increased in patients with grade 2 diastolic dysfunction (p = 0.04), whereas no association between RAP or RAP/PAWP ratio and diastolic function was found at rest.

**Table 3:** Hemodynamic measurements and calculations according to LV diastolic function at rest and during maximal exercise.

	REST			EXERCISE			P-VALUE*
	Normal	DD-1	DD-2	Normal	DD-1	DD-2	Prest/Pmax
**RAP, mmHg**	7±2	5±2	7±5	10±4	12±5	14±5	0.28/0.17
**PAWP, mmHg**	11±3	11±5	13±3	29±4	31±7	34±6	0.28/0.13
**PAPsys, mmHg**	25±8	28±6	33±3	58±14	66±11	74±9	0.02/0.01
**HR, bpm**	59±11	69±9	63±8	125±24	117±17	111±16	0.03/0.25
**RVSVi, ml/m^2^**	48±6	39±7	48±8	62±16	53±10	59±10	0.001/0.15
CI, l/(min*m^2^)	2.8±0.5	2.7±0.6	3.0±0.7	7.6±1.8	6.1±1.1	6.5±1.0	0.33/0.02
**Calculations**							
**PVR, wood**	1.1±0.6	1.3±0.5	1.3±0.4	1.3±0.5	1.4±0.5	1.4±0.3	0.44/0.69
RVSWi, g/(m^2^*beat)	6.7±2.5	6.8±2.2	9.1±3.7	29.6±7.9	24.9±6.3	29.8±7.4	0.07/0.11
**PAC, ml/mmHg**	7.1±2.7	5.5±2.3	5.6±1.1	2.6±0.8	1.9±0.7	2.0±0.5	0.05/0.06
**PAE, mmHg/ml**	0.3±0.1	0.4±0.1	0.7±0.2	0.5±0.2	0.7±0.2	0.7±0.2	0.01/0.04
**PAPi**	2.3±0.9	3.6±2.2	4.0±2.9	2.9±1.4	3.4±1.5	3.1±1.3	0.15/0.71
**RAP/PAWP**	0.6±0.1	0.5±0.2	0.5±0.4	0.3±0.1	0.4±0.1	0.4±0.2	0.38/0.58

Data are shown as mean±SD.

CI = cardiac index; HR = heart rate; PAP = pulmonary artery pressure; PAC = pulmonary artery compliance; PAPi = pulmonary artery pulsatility index; PAWP = pulmonary artery wedge pressure; PAE = pulmonary artery elastance; PVR = pulmonary vascular resistance; RAP = right atrial pressure; RVSWi = RV stroke work index; SVO_2_ = saturation mixed venous blood; SVR = systemic vascular resistance.

*P-value ANOVA. Differences in parameters according to diastolic function at rest and at max exercise (Prest/Pmax).

During supine exercise, patients achieved a median exercise load of 100 (81–119) Watts with no difference between grades of diastolic function (p = 0.87). Both RV and LV filling pressure increased significantly in all patients during exercise and PAWP increased above 30 mmHg after maximal exercise in 20 patients (49%). PAWP >30 mmHg was common in patients with grade 2 diastolic dysfunction (9 patients, 82%) compared to those with normal diastolic function (3 patients, 30%) (p = 0.03). An increase in mean RAP to ≥15 mmHg following exercise was not seen in patients with normal LV filling, compared to 4 patients (20%) with mild and 7 patients (63%) with moderate diastolic dysfunction (p = 0.003). However, RAP/PAWP was not different between groups. Further, systolic PAP was higher in patients with diastolic dysfunction after exercise.

**Fig 2:**
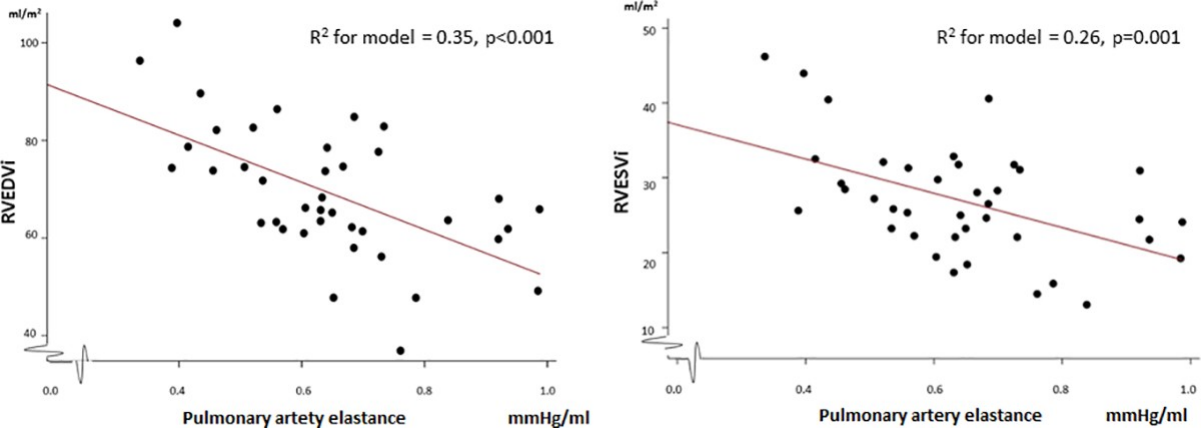
Association between RV end-diastolic and end-systolic volume indices and pulmonary artery elastance at maximal exercise. RVEDVi = right ventricular end-diastolic volume index, RVESVi = right ventricular end-systolic volume index.

At maximal exercise, PA elastance was increased in patients with diastolic dysfunction and showed an inverse relation with RV size at rest (RV end-diastolic volume index r = -0.59, p<0.001; RV end-systolic volume index r = -0.51, p = 0.001) (Fig 2). No association between RV stroke work index/PA pulsatility index and diastolic function was found at peak exercise.

**Fig 3:**
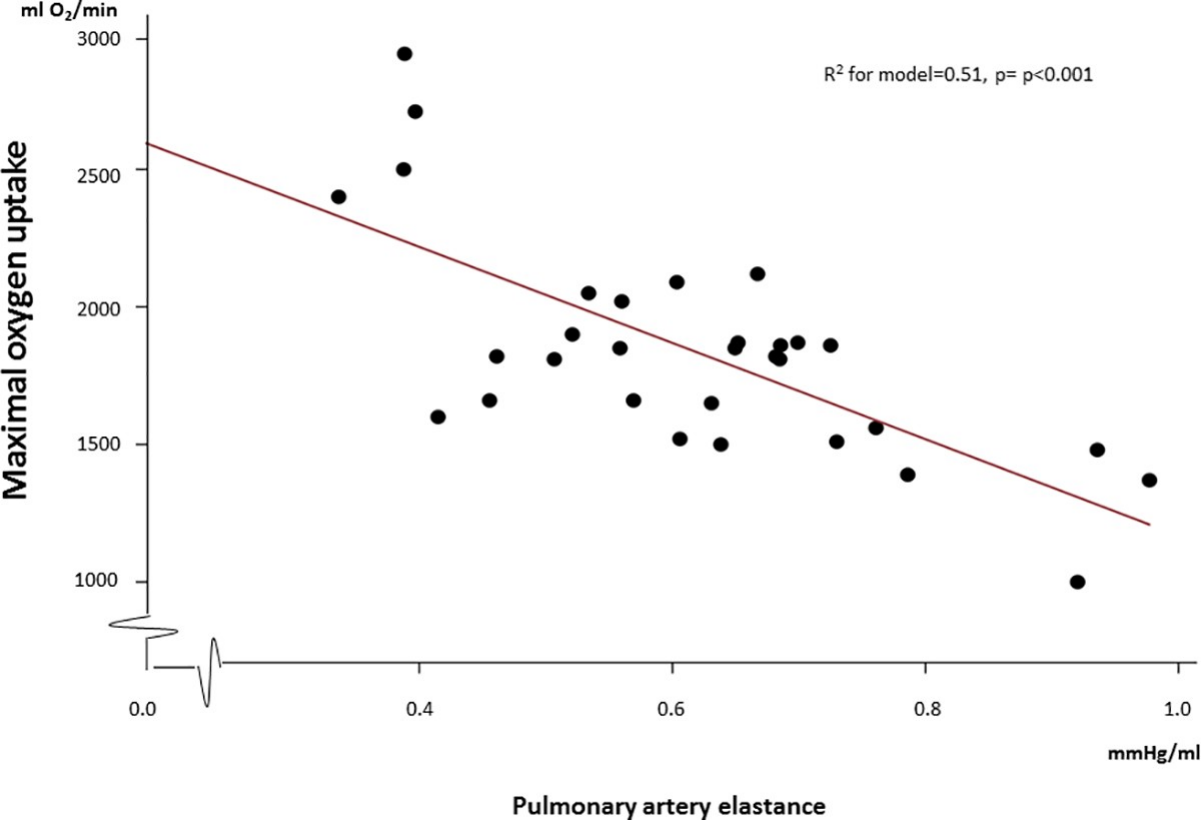
Association between maximal oxygen consumption and pulmonary artery elastance at maximal exercise.

Both PA elastance (r = -0.71, p<0.001) and PA compliance (r = 0.63, p<0.001) was associated with maximal oxygen consumption (Fig 3). After adjustment for cardiac output and PAWP at peak exercise, the association between PA elastance and maximal oxygen consumption remained significant (β = -0.40, p = 0.04).

## Discussion

The present study confirms the following two findings: (1) abnormal LV diastolic function is a common finding in asymptomatic patients with significant AS; (2) right ventricular afterload is increased when diastolic dysfunction is moderate. The increase in RV afterload is compensated at rest but is associated with increased right atrial pressure and inversely related to oxygen consumption during exercise. Morphologically, RV remodeling in patients with diastolic dysfunction was characterized by smaller chamber size suggesting reduced RV compliance in these patients.

AS is associated with excessive afterload during LV ejection. The classic LV response to normalize increased systolic wall stress on the myocardial sarcomere is myocardial hypertrophy, in which increased wall thickness counterbalances increased end-systolic pressure and end-systolic wall stress. Although initially an adaptive response, this will cause relaxation abnormalities and eventually lead to increased myocardial stiffness due to diffuse fibrosis causing LV diastolic dysfunction. We found that only one third of patients with asymptomatic significant AS had normal diastolic function. Previous studies have shown that the presence of diastolic dysfunction, especially when moderate or severe, was associated with severe symptoms and higher 1-year mortality than symptomatic AS [19]. The severity of diastolic dysfunction has also been associated with a poor outcome following aortic valve replacement in patients with severe AS [20, 21].

A hallmark of diastolic dysfunction is an increase in LV filling pressure especially during exercise [22, 23], which has also been demonstrated in patients with AS [7, 8]. In severe AS, the RV also experiences increased afterload due to postcapillary pulmonary artery hypertension. Further, as RV and LV share the interventricular septum, remodeling of the LV with increased wall thickness invariably affects the RV. Preclinical studies in canine hearts have shown that release of an aortic constriction in diastole mediates an abrupt decrease in LV systolic pressure, with a subsequent reduction in RV systolic pressure through ventricular systolic interdependence [6]. Studies have also demonstrated that increased filling pressure of one ventricle decreases compliance in the adjacent ventricle [24, 25]. Further, a recent study by Rain and colleagues elegantly demonstrated higher RV diastolic dysfunction due to increased chamber stiffness and sarcomere tension in patients with idiopathic pulmonary hypertension [26]. Taken together, both ventricular interaction and reduced RV compliance likely contributes to advanced diastolic dysfunction.

We found RV size was lower in patients with diastolic dysfunction. Given that the physiology of LV diastolic dysfunction in AS shares many similarities with HFpEF, we speculate that the small RV cavity with reduced compliance possibly signifies a distinct phenotype among asymptomatic AS patients.

At rest, the RV preload in terms of RAP was normal irrespective of LV diastolic function. However, during exercise, the RV preload increased considerably in patients with moderate diastolic dysfunction. In about 2/3 of the patients with diastolic dysfunction, RAP increased to 15mmHg or more while no increase was observed in patients with normal diastolic function. It is likely that patients with moderate diastolic dysfunction were not able to accommodate the increased venous return during exercise due to the increase in RAP.

Although RV preload, elastance, and compliance were affected, no signs of RV systolic dysfunction (in terms of TAPSE, S’, RVEF on cMRI, inadequate increase in RV stroke volume with exercise, RAP/PAWP ratio or PA pulsatility index) was observed. Thus, the observed changes in central hemodynamics were not associated with overt RV failure. There was a trend towards accentuated RV systolic function in patients with moderate diastolic dysfunction, which is in agreement with the study by Rain and colleagues where RV diastolic dysfunction was associated with an increase in RV contractility [26].

### Limitations

Grading of diastolic function is widely done using Doppler echocardiography, but grading systems have changed considerably over the last decade. In addition, the different grading systems have several inherit limitations and are not interchangeable. Since the patient population included in the present analysis was selected based on the ability to perform supine exercise test and excluded patients with atrial fibrillation and other cardiopulmonary disorders, likely resulted in the selection of a relatively healthy AS population. Further, the present study is not applicable for patients with symptomatic AS and more advanced co-morbidity as it included completion of maximal exercise testing, which according to guideline recommendations excludes symptomatic patients. Even though, there were no cases with significant anemia, the presence of patients with comorbidities including diabetes (N = 5) and coronary artery disease (N = 3) may have biased the results. Further, no systematic assessment of asymptomatic coronary artery disease was done using coronary angiography; thus the number of patients with significant coronary stenosis is unknown. Notably, all patients underwent maximal exercise testing without significant angina or development of new regional wall motion abnormalities on 2D echocardiography. Exercise was done in a semi-supine position, which is known to increase preload conditions compared with upright exercise. Since all patients performed exercise testing in a semi-supine position, it is unlikely to be the primary determinant of between-group differences but may have increased RAP in some patients. It would have been preferable to include T1 mapping measurements of diffuse myocardial fibrosis but was not performed due to non-availability at our institution during the study, and we were only able to complete assessment of focal fibrosis. The lack of association between focal fibrosis and diastolic function may be due to the small sample size; therefore the risk of type 2 error exists.

## Conclusion

The present study demonstrates that abnormal LV diastolic function is a common finding in asymptomatic significant AS, and when diastolic dysfunction is moderate, right ventricular afterload is increased. The increase in RV afterload is compensated at rest with no signs of right heart failure but is associated with increased right atrial pressure and inversely related to oxygen consumption during exercise. Morphologically, RV remodeling in patients with diastolic dysfunction was characterized by smaller chamber size suggesting reduced RV compliance.
